# Mutations in Rb1 pathway-related genes are associated with poor prognosis in Anaplastic Astrocytomas

**DOI:** 10.1038/sj.bjc.6602661

**Published:** 2005-06-21

**Authors:** L M Bäcklund, B R Nilsson, L Liu, K Ichimura, V P Collins

**Affiliations:** 1 Department of Oncology-Pathology, Karolinska Institutet, Karolinska University Hospital, SE-171 76 Stockholm, Sweden; 2 Department of Pathology, Division of Molecular Histopathology, University of Cambridge, Addenbrooke's Hospital, Box 231, Cambridge CB2 2QQ, UK

**Keywords:** survival, glioma, p53 pathway, Rb1 pathway, *PTEN*, *EGFR*, prognosis

## Abstract

Anaplastic astrocytoma (AA, WHO grade III) is, second to Glioblastoma, the most common and most malignant type of adult CNS tumour. Since survival for patients with AA varies markedly and there are no known useful prognostic or therapy response indicators, the primary purpose of this study was to examine whether knowledge of the known genetic abnormalities found in AA had any clinical value. The survival data on 37 carefully sampled AA was correlated with the results of a detailed analysis of the status of nine genes known to be involved in the development of astrocytic tumours. These included three genes coding for proteins in the p53 pathway (*TP53, p14*^*ARF*^ and *MDM2*), four in the Rb1 pathway (*CDKN2A, CDKN2B, RB1* and *CDK4*) and *PTEN* and *EGFR*. We found that loss of both wild-type copies of any of the three tumour suppressor genes *CDKN2A*, *CDKN2B* and *RB1* or gene amplification of *CDK4*, disrupting the Rb1 pathway, were associated with shorter survival (*P*=0.009). This association was consistent in multivariate analysis, including adjustment for age (*P*=0.013). The findings suggest that analysis of the genes coding for Rb1 pathway components provides additional prognostic information in AA patients receiving conventional therapy.

Adult astrocytomas are classified by WHO (Kleihues and Cavanee, 2000) into Diffuse Astrocytoma malignancy grade II (AII), Anaplastic Astrocytoma malignancy grade III (AA) and Glioblastoma malignancy grade IV (GB). Standard treatment of both AA and GB is surgery followed by radiotherapy. Anaplastic Astrocytoma patients have variable survival with a median survival of approximately 3 years – intermediate between AII (median approximately 7 years) and GB (8–12 months) (Kleihues and Cavanee, 2000; Herfarth *et al*, 2001; Backlund *et al*, 2003). The histological criteria for an AA diagnosis are also intermediate between the AII and the GB, the histological findings in AA ranging from tumours that may be difficult to separate from an AII to those that have to be distinguished from a GB. The fact that AA may develop from an AII or may arise *de novo* and that both have a tendency to develop into a GB also provides us with diagnostic difficulties. Adequate histological sampling is essential to exclude a GB. As with all astrocytic tumours, young age has been generally accepted as a positive prognostic factor for AA (Perry *et al*, 1999; Smith *et al*, 2001; Rasheed *et al*, 2002). However, no dependable histological criteria have been found to provide accurate prognostic indicators permitting a subdivision of AA into good and bad prognostic groups.

More than a dozen genes have been documented as being aberrant in astrocytic tumours and these abnormalities generally accumulate in a particular order in the different malignancy grades. *TP53* is the most commonly aberrant gene in AII and AA – approximately 50% of these tumours have no wild-type *TP53* and a further 10–15% have one mutated and one wild-type allele (Rasheed *et al*, 1994; Ichimura *et al*, 2000; Kato *et al*, 2000). Glioblastomas that develop from AII or AA also have frequent *TP53* mutations, while the incidence of *TP53* mutations in primary or *de novo* GB is lower – around 35% (Watanabe *et al*, 1996). Yet, primary GBs show disruption of the p53 pathway at similar levels to AII and AA due to homozygous deletions of *p14*^*ARF*^ or amplification and overexpression of *MDM2* (Ichimura *et al*, 2000). The majority of GBs also have genetic abnormalities disrupting the Rb1 pathway, that is, by one of the following: loss of wild-type *CDKN2A* (generally together with loss of wild-type *CDKN2B* by homozygous deletion) or loss of wild-type *RB1* or amplification and overexpression of *CDK4*. In addition, losses of wild-type *PTEN* occur in almost one-half of GB and amplification of *EGFR* in approximately one-third (Ueki *et al*, 1996; Rasheed *et al*, 1997; Schmidt *et al*, 1999; Ichimura *et al*, 2000). Anaplastic Astrocytoma also occasionally show losses of both wild-type copies of the three tumour suppressor genes (TSGs) located on 9p, *CDKN2A*, *CDKN2B*, *p14*^*ARF*^ (Ichimura *et al*, 2000), simultaneously disrupting both the Rb1 and p53 pathways, as well as loss of both wild-type copies of the *PTEN* gene on 10q (Schmidt *et al*, 1999). Loss of both wild-type *RB1* has not been reported in AA (Ueki *et al*, 1996; Ichimura *et al*, 2000). Amplification of proto-oncogenes occurs almost exclusively in GB. In AA, *CDK4 / MDM2* amplification is rarely seen and *EGFR* amplification occurs in <10% of the cases (Bigner *et al*, 1988; Ichimura *et al*, 2000). Thus, AAs as defined by histological criteria appear to consist of a small fraction showing genetic changes similar to GB (e.g. loss of wild-type *CDKN2A*, *CDKN2B*, *p14*^*ARF*^ and *PTEN*; amplification of *CDK4* and *EGFR*) and a majority showing abnormalities similar to AII (i.e. loss of wild-type *TP53*). In AA, the frequency of hemizygous deletions of significant gene loci is greater than that found in AII, but less than that found in GB and in most cases the retained allele is wild type (Schmidt *et al*, 1994; Nishizaki *et al*, 1998; Ichimura *et al*, 2000).

Attempts to correlate the genetic findings in AA with patient outcome have to-date not provided any convincing correlations with survival (Galanis *et al*, 1998; Olson *et al*, 1998; James *et al*, 1999; Smith *et al*, 2001; Rasheed *et al*, 2002).

The primary purpose of this study was to explore potential associations between somatic mutations and survival in a series of 37 AA, since the identification of genetic prognostic indicators would be of clinical value. We have studied three genes involved in the p53 pathway (*TP53*, *MDM2* and *p14ARF*), four genes involved in the Rb1 pathway (*RB1*, *CDKN2A*, *CDKN2B* and *CDK4*), *PTEN* and *EGFR*. With the exception of *TP53*, all these genes are more commonly altered in GB than in AA. Clinical data were also considered and tested for associations with survival and when appropriate used to adjust for potential confounders. Our findings suggest that patients with well-sampled tumours fulfilling the WHO criteria for AA (Kleihues and Cavanee, 2000) having genetic abnormalities affecting any of the genes coding for the components of the Rb1 pathway have a poor outcome.

## MATERIALS AND METHODS

### Patients and tumour tissue

A total of 37 AA patients operated at the Karolinska Hospital, Stockholm, or Sahlgrenska University Hospital, Gothenburg between 1989 and 1994 were included in the study. All tumours were extensively sampled for histology and were reassessed and classified as AA according to the histological criteria in the 2000 WHO classification (Kleihues and Cavanee, 2000). End of follow-up of the patients was set to 1st October 2003, providing a minimum follow-up time of 10.3 years. The clinical data were collected from the patient records at the hospitals where they were treated and / or followed up. The data included age and sex of the patient, tumour localisation, duration and type of symptoms before diagnosis, date of operation(s), postoperative radiotherapy and chemotherapy and date of death. Data were missing from a maximum of one case for any clinical parameter. All operations were gross total. Thus, no cases diagnosed on biopsy only were included. The tumour tissue was particularly well sampled – often all tissue was processed with adjacent pieces alternatively frozen for molecular analysis and fixed for histology (up to 15 blocks). The criteria for an AA diagnosis were strictly according to WHO (Kleihues and Cavanee, 2000). The tumours were moderately to highly cellular and the tumour cells showed a high nuclear : cytoplasmic ratio, distinct nuclear and cytoplasmic pleomorphism with coarse nuclear chromatin and mitotic activity. There was no notable microvascular proliferation and no necrotic foci found anywhere in the material from any case. All patients gave informed consent and ethics committee approval for the project was obtained at all sites.

### Genetic analysis

**Figure 1:**
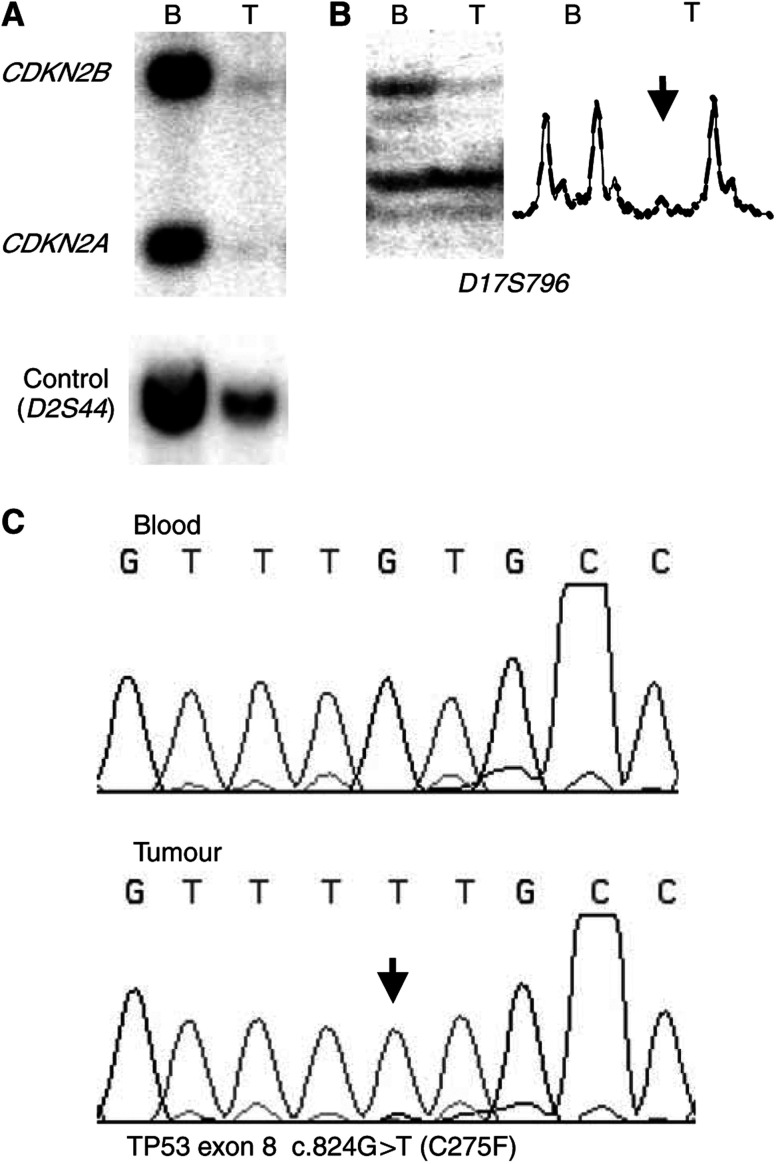
Genetic analysis of AA18. (**A**) A Southern blot hybridised with probes for *CDKN2A* and *CDKN2B* showing homozygous deletion of both genes. The control locus hybridisation (D2S44) on the same blot gives a clear signal from the tumour lane. (**B**) Microsatellite analysis at D17S796, the locus approximately 1.3 Mb telomeric to *TP53*. The densitometric profile indicates LOH in tumour DNA (arrow). (**C**) Sequencing of *TP53* exon 8. G at position 824 is mutated to T (arrow), resulting in Cys to Phe missense mutation at codon 275. B, blood DNA; T, tumour DNA.

Most of the genetic data on this tumour series has been previously published (Reifenberger *et al*, 1994; Ichimura *et al*, 1996; Schmidt *et al*, 1999; Ichimura *et al*, 2000; Liu *et al*, 2000). Briefly, tumour DNA was isolated from tumour pieces histopathologically judged to contain more than 70% tumour cells. Each tumour was genetically analysed and compared with the individual patient's white blood cell DNA. *RB1*, *CDKN2A*, *CDKN2B*, *p14*^*ARF*^, *TP53* and *PTEN* were analysed for deletions and mutations. The allelic status was assessed by studying paired samples of each patients’ blood and tumour DNA by either densitometry on Southern blotting using ^32^P-labelled gene-specific probes or microsatellite analysis using ^33^P-labelled PCR in loci in or around each gene by means of a PhosphorImager analysis (for details, see (Schmidt *et al*, 1994; Ichimura *et al*, 1996; Schmidt *et al*, 1999; Ichimura *et al*, 2000)). In addition, multiplex PCR was used for assessment of homozygous deletion of *PTEN*, as other methods would be complicated by the pseudogene of *PTEN* (Schmidt *et al*, 1999). Mutation screening of *RB1*, *CDKN2A*, *CDKN2B*, *p14*^*ARF*^, *TP53* and *PTEN* was carried out using either single-strand conformational polymorphism (SSCP), denaturing gradient gel electrophoresis (DGGE) or direct sequencing to identify somatic mutations in retained alleles (Ichimura *et al*, 1996; Schmidt *et al*, 1999; Ichimura *et al*, 2000) (see Figure 1). The presence or absence of amplification of the proto-oncogenes *CDK4*, *MDM2* and *EGFR* was determined using microsatellite analysis and Southern blotting with densitometry (Reifenberger *et al*, 1994; Ichimura *et al*, 2000; Liu *et al*, 2000). The genetic data were complete. In Table 2 each single patient is presented with an individual publication number, the same as used in previous publications (Reifenberger *et al*, 1994; Schmidt *et al*, 1994; Ichimura *et al*, 1996; Ichimura *et al*, 1998; Schmidt *et al*, 1999; Ichimura *et al*, 2000; Liu *et al*, 2000).

### Statistical analysis and definitions of genes and pathways as ‘normal’ or ‘abnormal’

The molecular information was restricted to genetic data. All genes studied were in each tumour classified as either ‘normal’ or ‘abnormal’. The TSGs (*CDKN2A*, *CDKN2B*, *RB1*, *TP53*, *p14*^*ARF*^ and *PTEN*) were categorised as ‘abnormal’ when there was no wild-type allele present, either due to homozygous deletion or deletion of one allele and mutation of the other. A comparison of survival in cases with all genes categorised as ‘normal’ with those having any gene(s) categorised as ‘abnormal’ was also carried out.

For the proto-oncogenes (*EGFR, CDK4* and *MDM2*), gene amplification (as in previous publications defined as a minimum average of five copies per genome) (Ichimura *et al*, 2000; Liu *et al*, 2000) was categorised as ‘abnormal’. Cellular pathways (p53 and Rb1 pathways) were categorized in a similar manner. A pathway was classified as ‘abnormal’ if *any* gene coding for a protein component of the pathway was classified as ‘abnormal’.

Of the 37 tumours analysed, 25 (68%) were from the patients’ first operation. They are referred to as ‘primary’ cases. A total of 12 patients (32%) had a prior operation and a histological diagnosis of either AII (six cases) or AA (six cases). The six cases that had a recurrent AA are referred to as ‘recurrent’ cases, while the six cases that had progressed from an AII are referred to as ‘progressed’ cases. The starting point for patient follow-up was the date of the operation from which the tumour tissue used in the genetic analysis was collected, survival being calculated from this date as we do not know the genetic status of the tumour removed at earlier operations. To address the potential bias of including the six ‘recurrent’ patients calculating survival as above, separate, additional correlation analysis was performed, with survival of the six ‘recurrent’ patients calculated from the date of their first AA operation, whenever a statistically significant association with survival was found.

To study the potential influence of single clinical or genetic factors on patient survival, univariate analysis using Wilcoxon–Gehan statistic (Gehan, 1965) was performed, with the patients divided into two groups, except for the age and duration of symptoms factors. When analysing age and survival, with age as a continuous variable, Cox Regression analysis was used. In multivariate analysis of the genetic factors and survival, Cox Regression analysis was carried out adjusting for age and whether the tumour studied was from a ‘primary’ or not (with the patients divided into two groups: ‘primary’ or ‘recurrent’ / ‘progressed’). All statistical analyses were performed using SPSS (Statistical Package for the Social Sciences) for Windows, release 12.0.1.

## RESULTS

### Clinical data

In Table 1, the clinical data for all cases are summarised with median survival and some statistical comparisons. In Table 2, the patients have been placed in one of three groups: (a) ‘primary’ AA; (b) ‘progressed’ AA (progressed from A) or (c) ‘recurrent’ AA. Postoperative survival in the whole series varied from 4 months to at least 13.9 years with a median of 4.3 years. Nine of the 37 patients were still alive at the end of follow-up, with a minimum follow-up time of 10.3 years. The fraction of males was uncommonly small in the series, with a male : female ratio of 1 : 1.5. There was no significant difference in survival between the sexes, or between the 33 (89%) patients with supratentorial and the four (11%) with infratentorial tumours. Seven (28%) of the 25 patients with ‘primary’ AA were alive at the end of follow-up as well as one (17%) of the six patients from each of the ‘recurrent’ and ‘progressed’ groups. The tumours were equally distributed to the left and right sides, with the patients with the tumour on the left side having shorter median survival, but only of borderline significance (*P*=0.078).

**Figure 2:**
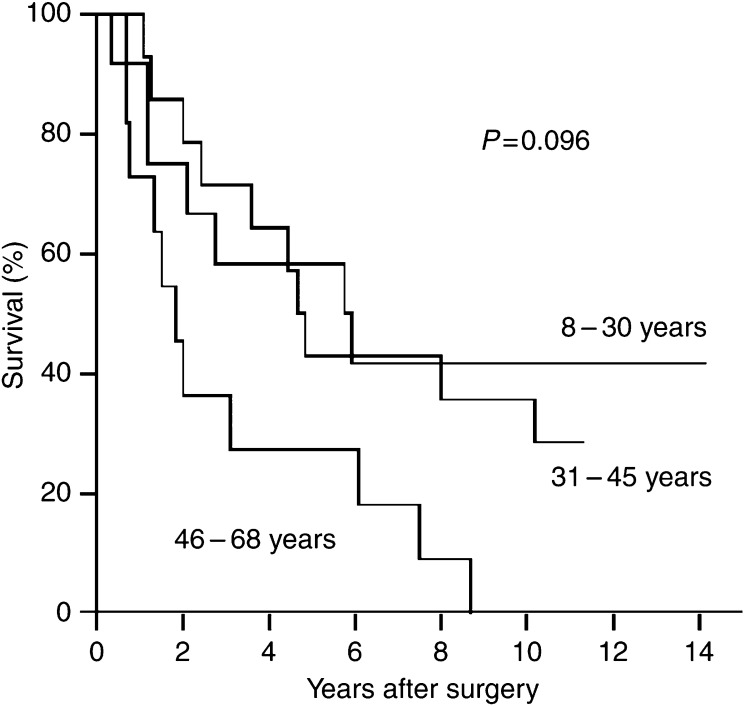
Survival curves in three different age groups: 8–30 years (*n*=12); 31–45 years (*n*=14); 46–68 years (*n*=11). Univariate analysis: *P*=0.096.

The mean age at the operation from which material was obtained was 38.8 years. The mean age at first diagnosis of AA in all the 37 patients was 37.3. Dividing all the 37 patients into three approximately equally large age groups (see Table 1 and Figure 2) demonstrates that the oldest age group (>45 years) had the shortest median survival (1.8 years), although with a wide range (220 days to 8.5 years). The association between age and survival was borderline significant both in univariate analysis of the three groups (*P*=0.096), and in Cox regression analysis with age as a continuous variable (*P*=0.093).

The most common début symptom in all cases indicating the disease process was epilepsy, described in 65% (24 out of 37) of the patients. The duration between these presenting symptoms and the operation for AA ranged in the ‘primary’ cases from a few days to one patient (AA59) with a history of epilepsy for 6.5 years. As shown in Table 1, dividing the ‘primary’ cases according to the duration of symptoms before operation revealed no significant survival differences.

In the following text, the cases will be referred to in the order that they appear in Table 2:

The 25 ‘primary’ AA had a median survival of 5.6 years and a mean age of 41 years. In all, 15 of them received postoperative radiotherapy (RT) at a total dose of 50–54 Gy given in 28–30 fractions over 6 weeks. One patient (AA29) was given a course of 37 Gy over 2.5 weeks and survived 1.9 years. Eight patients (AA59, AA76, AA26, AA65, AA73, AA105, AA94 and AA57) were never irradiated. In one case (AA106), it was not possible to access the postoperative notes. As shown in Table 1, the median postoperative survival of the eight ‘primary’ nonirradiated AA patients (6.3 years) was slightly, but not significantly, longer than for the 16 irradiated patients (5.1 years). Four of the 25 ‘primary’ AA patients (AA69, AA37, AA94, AA7) received chemotherapy postoperatively, one of which received polychemotherapy (AA69). Six of the 25 patients (AA102, AA69, AA100, AA79, AA37 and AA93) were later reoperated upon due to relapse and three of them (AA69, AA100 and AA93) then had evidence of progression to GB. The average time to progression in these three cases was 1.9 years.

Six patients had progressed from an AII to an AA (average time to progression to AA was 4.4 years). Their mean age was 32 years and median survival following the AA operation was 2.1 years. Three of them were irradiated after the AA operation (AA104, AA50 and AA3), while the other three (AA17, AA18 and AA86) had received postoperative RT after their first operation for AII. AA86 received postoperative chemotherapy and AA104 was reoperated again after 19 months at which time the tumour had progressed to a GB. AA3 was still alive at the end of follow-up (survival >13.4 years after AA and >13.8 years after the AII diagnosis).

The six ‘recurrent’ cases had had AA at the first operation and their mean age at that time was 28.5 years. Median survival, counted from that first AA operation, was 10.8 years while median survival from the operation for the recurrence was 1.6 years. Of the six ‘recurrent’ cases, three (AA2, AA87, AA51) had had postoperative RT after the first operation. AA53 received RT after the relapse and two patients (AA81 and AA13) did not receive RT at any time. AA81 was given postoperative chemotherapy after the recurrence, while AA51 had received chemotherapy after the first operation. Three patients (AA51, AA53, AA87) were reoperated a third time and none showed evidence of progression to GB.

### Genetic data

The genetic data for each individual tumour is presented in Table 2, with mutation nomenclature according to recommendations of the Nomenclature Working Group whenever applicable (Antonarakis, 1998). In Table 3, the results of univariate and multivariate analysis of the potential influence of single genetic factors on patient survival are given. In these analyses, shorter median survival was always seen in cases categorised as ‘abnormal’ as compared to those categorised as ‘normal’.

**Figure 3:**
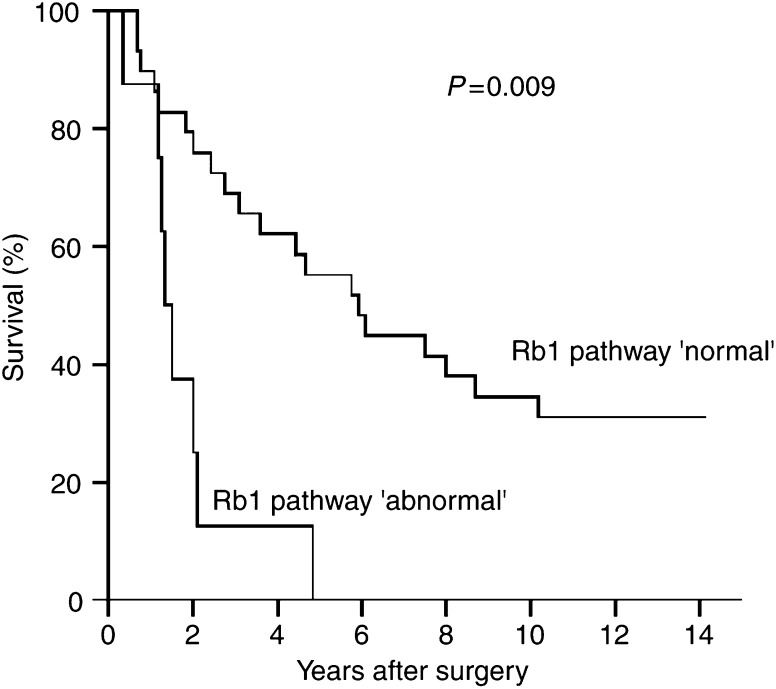
Survival curves for cases with loss of both wild-type alleles of any TSG coding for a component of the Rb1 pathway (*CDKN2A*, *CDKN2B*, *RB1*) or amplification of *CDK4*, that is, Rb1 pathway classified as ‘abnormal’ (*n*=8; median survival 1.4 years) and cases coded as ‘normal’ (*n*=29; median survival 5.8 years), respectively. Univariate analysis: *P*=0.009.

As presented in Table 3, the only single genetic aberration showing a significant negative association with survival was *CDK4* amplification. The statistical significance was borderline in univariate analysis (*P*=0.058) but significant in multivariate analysis adjusting for age and whether the tumour was ‘primary’ or ‘recurrent’ / ‘progressed’ (*P*=0.019). Disruption of the Rb1 pathway was negatively associated with survival in univariate (*P*=0.009) as well as in multivariate analysis (*P*=0.013). The survival curves for the Rb1 pathway groups ‘normal’ and ‘abnormal’ are shown in Figure 3.

A total of 15 cases (AA15, AA59, AA76, AA34, AA102, AA26, AA69, AA100, AA61, AA105, AA37, AA52, AA104, AA50, AA81) with all genes classified as ‘normal’ survived longer than those with at least one of the genes classified as ‘abnormal’. This was statistically significant in univariate analysis (*P*=0.013).

To avoid bias in including the survival data counting survival for the six ‘recurrent’ patients with the ‘recurrent’ operation as starting point, separate survival analyses were carried out when a statistically significant association between the genetic results and survival was found. As described in Materials and Methods, this was done calculating survival for these six cases from the date of their first AA operation. Similar *P*-values as those reported above were seen in each additional univariate and multivariate analysis. For the main finding, the association between Rb1 pathway and survival, the *P*-value in multivariate analysis was 0.014 (compared to *P*=0.013).

## DISCUSSION

We have focused exclusively on AA as defined by the latest edition of the WHO classification of tumours of the nervous system (Kleihues and Cavanee, 2000). Each tumour was extensively sampled for histology and no tumours with the histological characteristics of GB or AII were accepted for inclusion. Each piece of tissue used for the genetic analysis was histologically characterised to ensure that tumour tissue was studied. This was confirmed by the genetic analysis of the tumour tissue which, when compared with the individual patients’ white blood cell DNA, showed genetic abnormalities in all cases (e.g. loss of heterozygosity (LOH) at a number of sites in addition to point mutations) (Ichimura *et al*, 1998; Miyakawa *et al*, 2000). The age range of the patients and the location of the tumours are comparable to similar studies, while the sex distribution is slightly unusual showing comparatively high number of female subjects (Kleihues and Cavanee, 2000). A linear relationship between young age and longer survival was not possible to demonstrate with the limited number of patients in this study, but when the patients were divided up in three equally large groups, the subjects in the oldest group had the worst and the youngest age group the best outcome. The survival data overall in this series is in accord with previous studies (Perry *et al*, 1999; Herfarth *et al*, 2001; Lin *et al*, 2003) showing a huge variation from a few months to more than 13 years, widely overlapping that observed for both AII and GB. Our minimum follow-up here was 10.3 years, which is necessary in studies where survival can be long.

In a situation where no curative treatment is available, as is the case for AA, therapy is often modified individually unless all patients have been included in a particular clinical trial. Most, but far from all, patients received postoperative RT of 50–54 Gy. This variation in treatment is further complicated by the ‘recurrent’ and ‘progressed’ tumours, where the patients have received radiotherapy at their first diagnosis and were therefore not eligible for further irradiation. In addition, it is not unusual to use cytotoxic drugs at recurrences.

The primary aim of this study was to test for potential associations between mutations and survival in a clinically, histopathologically and genetically well-defined series of AA. There are few studies of this type where only AA is analysed. Most studies combine AA with the much more malignant GB under the terms ‘malignant astrocytomas’ or ‘malignant gliomas’. The study of Smith *et al* (2001) analysed AA separately and found a positive association between *TP53* mutation and survival and a negative association between loss of wild-type *PTEN* and survival. They included a large number of AA diagnosed with biopsy only, making a reliable histological classification more difficult. We did not find any clear associations between *TP53* mutation and survival in any of the comparisons. Since *TP53* mutations can have a dominant negative effect (Willis *et al*, 2004), we even performed an analysis with cases with *TP53* mutation and retention of one wild-type allele coded as ‘abnormal’, and this, too, showed no association with survival (*P*=0.165). It is notable that the commonest point mutation of *TP53* (while differing in base substitution) occurred at codon 273 in 27% of the tumours (Table 2). Similar findings have been reported in GB recently (Ohgaki *et al*, 2004).

As seen in Table 2, some tumours had loss of one wild-type allele of some TSGs. In fact, for all studied TSGs, this was relatively frequent. We generally do not know the biological significance of TSGs losing only one allele. It may result in lowered molar levels of the gene product providing a growth advantage. The concept of haploinsufficiency has been well documented for relatively few genes, but might be of relevance, for example, *CDKN2A* and *p14*^*ARF*^ (see review by Quon and Berns, 2001, and references therein) (Quon and Berns, 2001). Epigenetic mechanisms, such as methylation, could also be of importance. Although the sensitivity of SSCP and DGGE as mutation screening methods for point mutations is high, we cannot exclude the possibility that we have missed mutations in single cases, since sequencing analysis was only performed in cases where abnormalities were detected by SSCP or DGGE.

Although 22 of 37 AAs had loss of one *PTEN* allele, only one (AA29) had lost both wild-type *PTEN* copies (loss of one copy and point mutation of the stop codon of the other resulting in the addition of eight amino acids to the C-terminal). The biological consequence of this *PTEN* mutation is unclear as all critical regions of the protein were wild type. Survival was relatively short in this case (1.9 years). In a previous study of 129 GB, we found that a combination of aberrations of Rb1 pathway-related genes by loss of both wild-type alleles of TSGs or amplification of proto-oncogenes (almost always combined with p53 pathway abnormalities) and loss of both wild-type copies of *PTEN* was associated with particularly poor outcome (Backlund *et al*, 2003). It is important to note that none of the AAs here had this genetic profile.

The major finding of the present study is that patients with AA who have a somatic mutation profile resulting in loss of both wild-type copies of TSGs or amplification of oncogenes involved in the Rb1 pathway (as found frequently in GB), have a worse outcome as compared to AAs without these abnormalities. There was two out of 25 (8%) ‘primary’ AAs with this type of Rb1 pathway abnormality. The ‘recurrent’ and ‘progressed’ cases showed this type of Rb1 pathway abnormality in 50%. *TP53* mutations, generally with loss of the second allele, are much more common in our cohort of AA (about two-third of all cases regardless of whether ‘primary’ or ‘recurrent’) than we found in our GB series (45 out of 129 or 35%) (Backlund *et al*, 2003), a finding that is in line with previous reports (Rasheed *et al*, 1994; Kato *et al*, 2000).

The material examined and the findings of this study are complex. The numerous statistical tests performed in a retrospective study like this have to be interpreted with caution. A larger series of AA will have to be examined to confirm and extend these results. New developments are providing the technologies that will permit high throughput genetic analysis on larger series of uniformly treated patients. Hopefully, the findings in this study will stimulate further efforts in this regard leading to improved analysis of AA, providing clinicians with important prognostic information and eventually targets for molecular therapy.

## Figures and Tables

**Table 1:** Clinical data, median postoperative survival and statistical analysis

	**No. of cases**	**Median survival (years)**	***P*-value**
Overall postoperative survival (deceased 28; alive 9)	37	4.3	
Age in relation to survival (Cox Regression, with age as a continuous varaiable; Mean age: 38.8 years			*P*=0.093
			
Age groups (years) (Wilcoxon–Gehan, with patients divided into approximately equally large groups)			
8–30	12	5.7	
31–45	14	4.7	*P*=0.096^a^
46–68	11	1.8	
			
*Sex*			
Female	22	3.9	
Male	15	5.6	*P*=0.607^a^
			
*Localisation*			
Cerebral	33	4.3	
Infratentorial	4	>6.2	*P*=0.605^a^
			
*Side* b			
Left	17	2.3	
Right	17	6.0	*P*=0.078^a^
‘Primary’ cases^c^	25	5.6	
‘Recurrent’ and ‘progressed’ cases	12	2.0	*P*=0.113^a^^,^^c^
			
*Duration of symptoms prior to operation* d			
0–3 months	10	4.0	
>3–<12 months	7	5.8	*P*=0.628^a^
12 months –	7	8.5	
			
*Postoperative radiotherapy* d			
Yes	16	5.1	
No	8	6.7	*P*=0.100^a^

a Univariate analysis of postoperative survival using Wilcoxon–Gehan.

b Three cases with mid-line location not included in this analysis.

c For definitions of ‘primary’, ‘recurrent’ and ‘progressed’ cases, see Material and Methods.

d ‘Primary’ cases only (one missing data).

**Table 2:** Summary of clinical and genetic data^a^ on all 37 AA. Cases are grouped as ‘primary’, ‘progressed’ and ‘recurrent’ AA (for definitions, see Materials and Methods) and then in subgroups with similar genetic data, with shortest survival first in each subgroup

**Publication number**	**Male / female**	**Age (years)**	**Tumour localisation^b^**		**Postoperative RT (Gy)**	**Postoperative chemotherapy**	**Reoperated later**	**Survival (years)**	***CDKN2A***	***CDKN2B***	***RB1***	***CDK4***	***MDM2***	***TP53***	***p14^ARF^***	***EGFR***	***PTEN***
	‘Primary’ cases (*n*=25; median survival=5.6 years; mean age: 41 years)																
AA15	F	24	Supra	Left	54	No		5.8	+ / +	+ / +	+ / +	+ / +	+ / +	+ / −	+ / +	+ / +	+ / −
AA59	F	65	Supra	Right	0	No		8.5	+ / +	+ / +	+ / +	+ / +	+ / +	+ / +	+ / +	+ / +	+ / +
AA76	M	26	Infra	Central	0	No		>10.3	+ / +	+ / +	+ / +	+ / +	+ / +	+ / +	+ / +	+ / +	+ / +
AA34	M	38	Supra	Right	54	No		>11.1	+ / +	+ / +	+ / +	+ / +	+ / +	+ / +	+ / +	+ / +	+ / +
AA102	F	26	Infra	Central	50	No	Yes	>13.9	+ / −	+ / −	+ / −	+ / +	+ / +	+ / +	+ / −	+ / +	+ / −
AA26	F	67	Supra	Left	0	No		0.6	+ / +	+ / +	+ / −	+ / +	+ / +	+ / C176Y, R249S	+ / +	+ / +	+ / −
AA10	M	47	Supra	Right	54	No		0.7	+ / −	+ / −	+ / −	+ / +	+ / +	− / R282W	+ / −	+ / +	+ / −
AA65	M	12	Infra	Central	0	No		1.1	+ / +	+ / +	+ / −	+ / +	+ / +	− / R273H	+ / +	+ / +	+ / −
AA106	F	57	Supra	Right	nk^c^	nk^c^		1.8	+ / +	+ / +	+ / +	+ / +	+ / +	− / V197G	+ / +	+ / +	+ / −
AA69	F	8	Supra	Right	54	Yes	Yes	2.7	+ / +	+ / +	+ / +	+ / +	+ / +	+ / R273C	+ / +	+ / +	+ / +
AA73	M	62	Supra	Left	0	No		3.0	+ / −	+ / −	+ / +	+ / +	+ / +	− / R273L	+ / −	+ / +	+ / −
AA100	F	34	Supra	Right	50	No	Yes	3.5	+ / +	+ / +	+ / +	+ / +	+ / +	+ / R273C, T304-305ins	+ / +	+ / +	+ / +
AA101	F	35	Supra	Left	50	No		4.3	+ / +	+ / +	+ / −	+ / +	+ / +	− / R175H	+ / +	+ / +	+ / +
AA61	F	34	Supra	Left	54	No		4.6	+ / +	+ / +	+ / −	+ / +	+ / +	+ / R273C	+ / +	+ / +	+ / +
AA79	M	22	Supra	Right	50	No	Yes	5.6	+ / +	+ / +	+ / +	+ / +	+ / +	− / W146X	+ / +	+ / +	+ / +
AA105	M	50	Supra	Right	0	No		6.0	+ / +	+ / +	+ / −	+ / +	+ / +	+ / H193R	+ / +	+ / +	+ / −
AA37	M	35	Supra	Right	50	Yes	Yes	7.9	+ / −	+ / −	+ / +	+ / +	+ / +	+ / R273H	+ / −	+ / +	+ / +
AA94	F	68	Supra	Right	0	Yes		7.3	+ / +	+ / +	+ / +	+ / +	+ / +	− / R273C	+ / +	+ / +	+ / −
AA57	F	31	Supra	Left	0	No		>10.3	+ / +	+ / +	+ / +	+ / +	+ / +	− / R110L	+ / +	+ / +	+ / +
AA19	M	32	Supra	Right	54	No		>11.0	+ / +	+ / +	+ / +	+ / +	+ / +	− / E180K	+ / +	+ / +	+ / +
AA20	F	40	Supra	Left	54	No		>11.1	+ / +	+ / +	+ / +	+ / +	+ / +	− / R273C	+ / +	+ / +	+ / −
AA52	F	30	Supra	Right	54	No		>11.9	+ / +	+ / +	+ / +	+ / +	+ / +	+ / T102del	+ / +	+ / +	+ / −
AA7	F	59	Supra	Right	50	Yes		1.3	+ / −	+ / −	+ / +	+ / A	+ / +	+ / +	+ / −	+ / A	+ / −
AA93	F	57	Supra	Right	52	No	Yes	1.5	− / −	− / −	+ / +	+ / +	+ / +	+ / +	− / −	+ / A	+ / −
AA29	F	60	Supra	Left	37	No		1.9	+ / −	+ / −	+ / +	+ / +	+ / +	+ / +	+ / −	+ / +	− / X404S^d^
	‘Progressed’ cases (*n*=6; median survival=2.1 years; mean age: 32 years)																
AA104	M	35	Supra	Left	54	No	Yes	2,3	+ / +	+ / +	+ / +	+ / +	+ / +	+ / +	+ / +	+ / +	+ / −
AA50	M	42	Supra	Left	54	No		9,9	+ / +	+ / +	+ / +	+ / +	+ / +	+ / −	+ / +	+ / +	+ / −
AA3	M	23	Supra	Right	50	No		>13,4	+ / +	+ / +	+ / +	+ / +	+ / +	− / I254T	+ / +	+ / +	+ / +
AA17	F	27	Supra	Left	54^e^	No		0,3	+ / −	+ / −	+ / +	+ / A	+ / +	− / R273C	+ / −	+ / +	+ / −
AA18	M	18	Supra	Left	54^e^	No		1,1	− / −	− / −	+ / −	+ / +	+ / +	− / C275F	− / −	+ / +	+ / −
AA86	M	45	Supra	Right	nk^e^	Yes		2,0	− / N71K	+ / −	+ / −	+ / +	+ / +	− / K320del	− / L86V	+ / +	+ / −
	‘Recurrent’ cases (*n*=6; median survival=1.6 years; mean age: 38 years)																
AA81	F	30	Supra	Right	0	Yes		>11,0	+ / +	+ / +	+ / +	+ / +	+ / +	+ / +	+ / +	+ / +	+ / +
AA2	F	51	Supra	Left	50^e^	No		0,6	+ / −	+ / −	+ / −	+ / +	+ / +	− / R110del	+ / −	+ / +	+ / +
AA13	F	45	Supra	Left	0	No		1,0	+ / −	+ / −	+ / −	+ / +	+ / +	− / K164E	+ / −	+ / +	+ / +
AA87	M	43	Supra	Left	50^e^	No	Yes	1,2	− / −	− / −	+ / +	+ / +	+ / +	− / R273C	− / −	+ / +	+ / −
AA53	F	18	Infra	Left	54	No	Yes	2,0	− / −	− / −	+ / +	+ / +	+ / +	+ / +	− / −	+ / +	+ / −
AA51	F	39	Supra	Left	50^e^	Yes^f^	Yes	4,7	− / −	− / −	+ / +	+ / +	+ / +	− / I232T	− / −	+ / +	+ / −

a + / + =two wild-type alleles; + / − =loss of one allele; mutations of alleles replace ‘ − ‘ and are annotated as recommended (Antonarakis, 1998); + / A=amplification of one allele.

b Supra=supratentorial location; infra=infratentorial location.

c nk=postoperative therapy not known.

d Stop codon mutated leading to a prolonged protein by eight amino acids (SIFFYQEGX according to HUGO).

e Radiotherapy given postoperatively after a previous operation (nk=doses not known).

f Chemotherapy given postoperatively after a previous operation.

**Table 3:** Median postoperative survival in all cases with or without any gene or pathway classified as ‘abnormal’ (see Material and Methods). Univariate and multivariate analysis

	***CDKN2A***	***CDKN2B***	***RB1***	***CDK4***	**Rb1 pathway**	***TP53***	***p14^ARF^***	***MDM2***	**p53 pathway**	***PTEN***	***EGFR***	**All 9 genes^a^**
Median survival in years in ‘normal’ cases^b^	5.6	5.4	4.3	4.6	5.8	5.8	5.6	4.3	6.0	4.5	4.6	7.9
(*n*=)	(31)	(32)	(37)	(35)	(29)	(19)	(31)	(37)	(17)	(36)	(35)	(15)
												
Median survival in years in ‘abnormal’ cases^b^	17	1.5	–	0.8	1.4	2.5	1.7	–	2.0	1.9	1.4	1.9
(*n*=)	(6)	(5)	(0)	(2)	(8)	(18)	(6)	(0)	(20)	(1)	(2)	(22)
												
Wilcoxon-Gehan P=	0.081	0.112		0.058	0.009	0.165	0.081		0.053	0.540	0.199	0.013
Cox Regression^c^ P=	0.102	0.116		0.019	0.013	0.126	0.102		0.223	0.556	0.139	0.093

a Comparing survival of the cases with all nine genes categorised as ‘normal’ with those having at least one of the nine genes categorised as ‘abnormal’.

b For definitions of gene abnormalities and pathway abnormalities, see Materials and Methods.

c Multivariate analysis using Cox Regression adjusting for age and whether the tumour studied was from a ‘primary’ or ‘recurrent’ / ‘progressed’ case.
